# Supplementary figures and images for: Associations of lymphocyte subpopulations with clinical phenotypes and long-term outcomes in juvenile-onset systemic lupus erythematosus
Source: PLoS One. 2022 Feb 7;17(2):e0263536. doi: 10.1371/journal.pone.0263536 (PMC8820627; doi:10.1371/journal.pone.0263536)

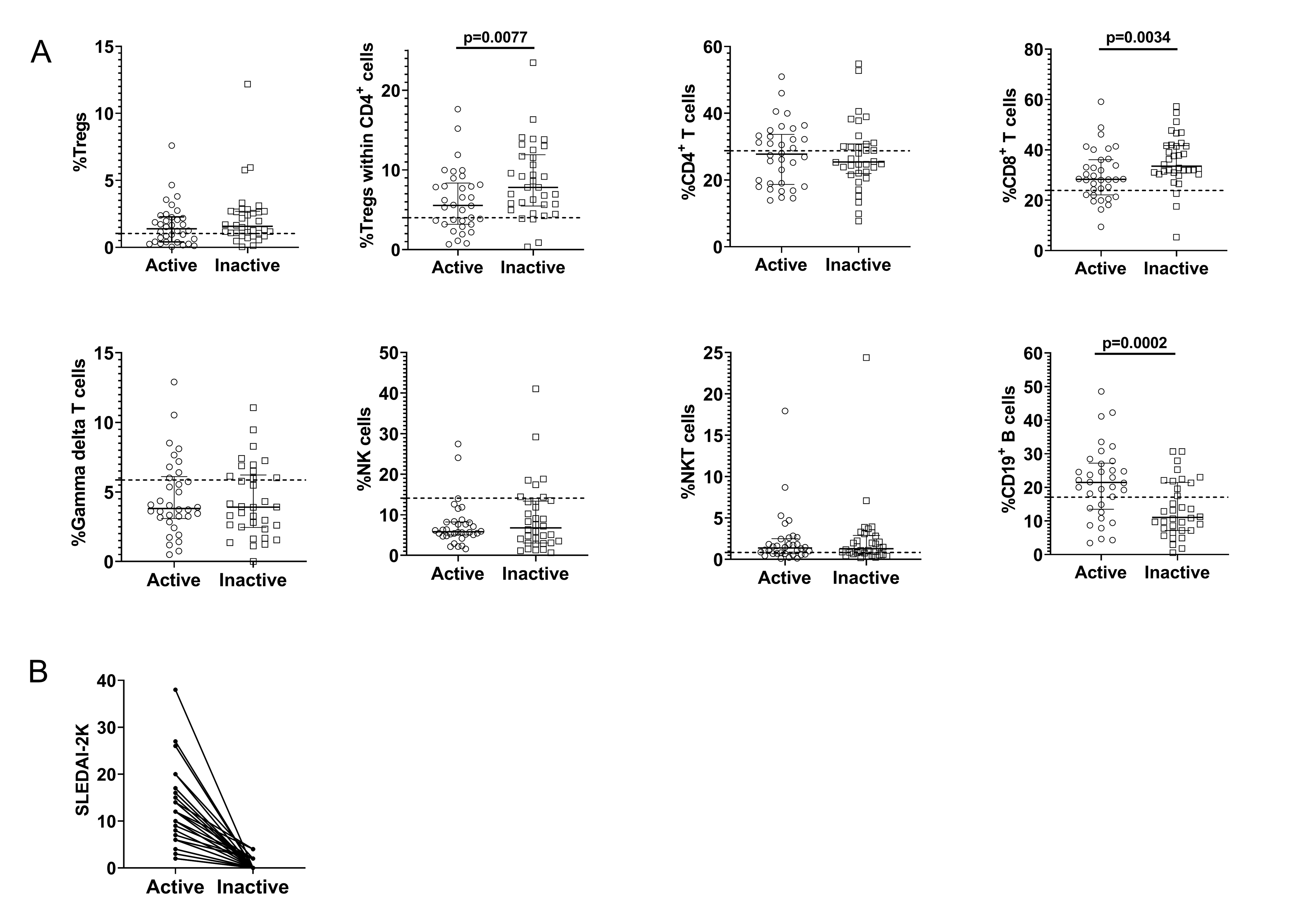

Supplement: S1 Fig — (A) Percentages of lymphocyte subsets in 34 JSLE patients during active disease and follow-up until inactive disease. (B) Disease activity measured by SLEDAI-2K during active disease and follow-up until inactive disease in 34 JSLE patients. Horizontal solid lines represent medians and interquartile ranges. The dotted horizontal lines in (A) show the medians for healthy controls. The connected lines in B represent individual patients. JSLE, juvenile-onset systemic lupus erythematosus; Tregs, regulatory T cells; NK cells, natural killer cells; NKT cells, natural killer T cells; SLEDAI-2K, systemic lupus erythematosus disease activity index 2000. (TIF) [file pone.0263536.s001.tif]

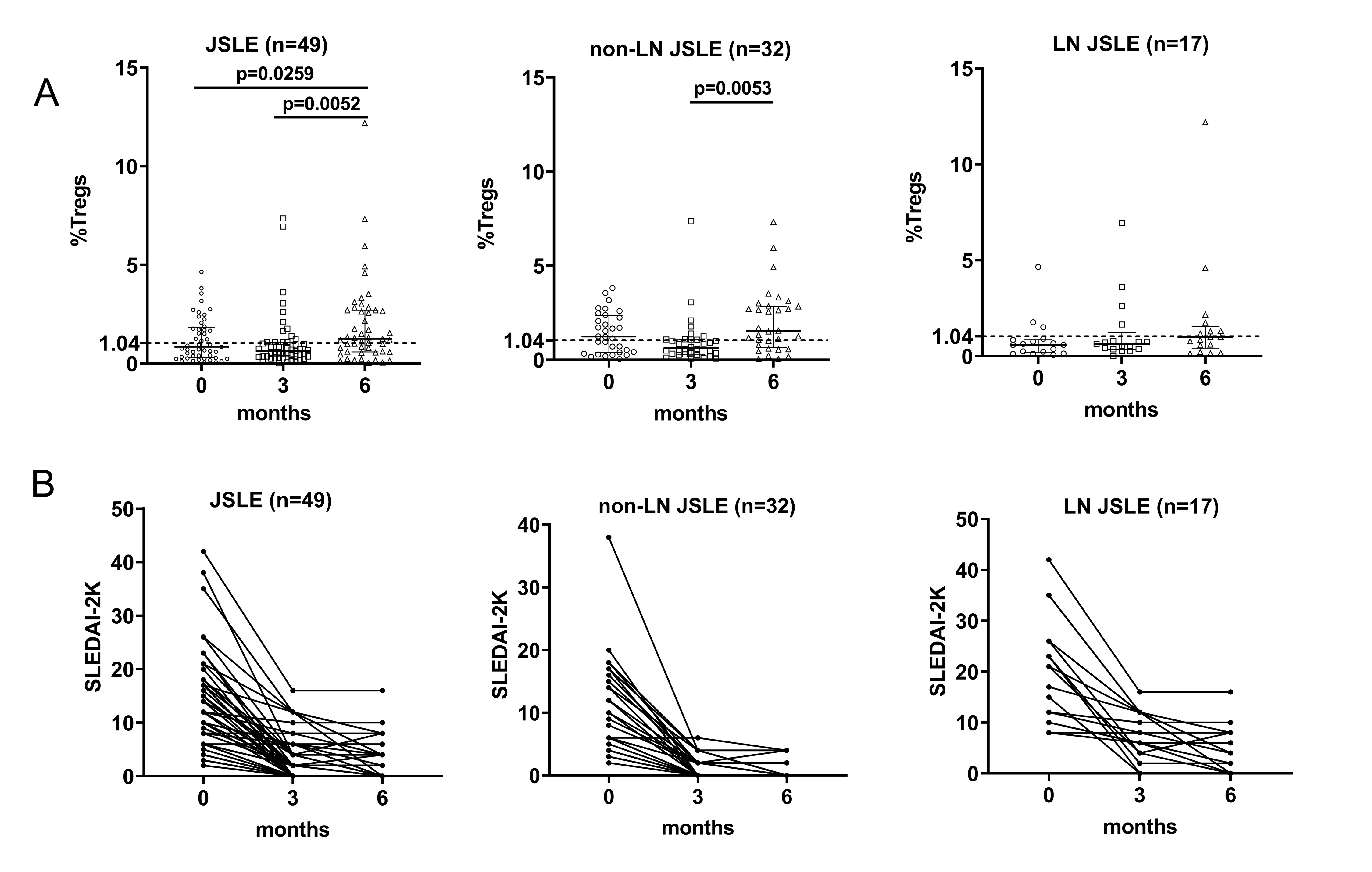

Supplement: S2 Fig — (A, B) Percentages of Tregs (A) and SLEDAI-2K (B) at 0, 3, and 6 months of follow-up in 49 JSLE patients. Horizontal lines represent medians and interquartile ranges. The horizontal dotted lines in (A) illustrate the medians in healthy controls. The connected lines in (B) represent individual patients. JSLE, juvenile-onset systemic lupus erythematosus; LN, lupus nephritis; Tregs, regulatory T cells; SLEDAI-2K, systemic lupus erythematosus disease activity index 2000. (TIF) [file pone.0263536.s002.tif]
