# Supplementary material for: Associations of lymphocyte subpopulations with clinical phenotypes and long-term outcomes in juvenile-onset systemic lupus erythematosus
Source: PLoS One. 2022 Feb 7;17(2):e0263536. doi: 10.1371/journal.pone.0263536 (PMC8820627; doi:10.1371/journal.pone.0263536)
Supplement: S1 Table — *A value of p<0.05 was considered to indicate statistical significance. Data are presented as percentages of patients. In the column, patients were stratified into 2 groups, high and low, based on the median percentage of each subset, and the presence or absence of each clinical presentation in the row. AIHA, autoimmune hemolytic anemia; LN, lupus nephritis; Tregs, regulatory T cells; NK cells, natural killer cells; NKT cells, natural killer T cells. (DOCX) [file pone.0263536.s003.docx]

| **Clinical manifestations** | **Lymphocyte subsets** | | | | | | | | | | | | | | | | | | | | | | | | | | |
| --- | --- | --- | --- | --- | --- | --- | --- | --- | --- | --- | --- | --- | --- | --- | --- | --- | --- | --- | --- | --- | --- | --- | --- | --- | --- | --- | --- |
|  | **CD4^+^ T cells** | | | **CD8+ T cells** | | | **γδ T cells** | | | **Tregs** | | | **Tregs/CD4+ cells** | | | | **CD19+ B cells** | | | | **NK cells** | | | | **NKT cells** | | |
|  | **Low** | **High** | **p-value** | **Low** | **High** | **p-value** | **Low** | **High** | **p-value** | **Low** | **High** | **p-value** | **Low** | **High** | **p-value** | **Low** | | **High** | **p-value** | **Low** | | **High** | **p-value** | **Low** | | **High** | **p-value** |
| Fever (-) | 80 | 58.6 | 0.08 | 62.1 | 75.9 | 0.26 | 73.3 | 64.3 | 0.46 | 66.7 | 70 | 0.78 | 65.5 | 71 | 0.65 | 79.3 | | 58.6 | 0.09 | 65.5 | | 72.4 | 0.57 | 75.9 | | 62.1 | 0.26 |
| Fever (+) | 20 | 41.4 |  | 37.9 | 24.1 |  | 26.7 | 35.7 |  | 33.3 | 30 |  | 34.5 | 29 |  | 20.7 | | 41.4 |  | 34.5 | | 27.6 |  | 24.1 | | 37.9 |  |
|  |  |  |  |  |  |  |  |  |  |  |  |  |  |  |  |  | |  |  |  | |  |  |  | |  |  |
| Skin (-) | 46.7 | 31 | 0.22 | 37.9 | 41.4 | 0.79 | 46.7 | 32.1 | 0.26 | 43.3 | 33.3 | 0.43 | 41.4 | 35.5 | 0.64 | 34.5 | | 44.8 | 0.42 | 41.4 | | 37.9 | 0.79 | 37.9 | | 41.4 | 0.79 |
| Skin(+) | 53.3 | 69 |  | 62.1 | 58.6 |  | 53.3 | 67.9 |  | 56.7 | 66.7 |  | 58.6 | 54.5 |  | 65.5 | | 55.2 |  | 58.6 | | 62.1 |  | 62.1 | | 58.6 |  |
|  |  |  |  |  |  |  |  |  |  |  |  |  |  |  |  |  | |  |  |  | |  |  |  | |  |  |
| Mucosal ulcer (-) | 63.3 | 44.8 | 0.15 | 44.8 | 62.1 | 0.19 | 73.3 | 32.1 | 0.003* | 53.3 | 53.3 | 1.0 | 55.2 | 51.6 | 0.78 | 55.2 | | 51.7 | 0.79 | 69 | | 37.9 | 0.03* | 48.3 | | 58.6 | 0.43 |
| Mucosal ulcer (+) | 36.7 | 55.2 |  | 55.2 | 37.9 |  | 26.7 | 67.9 |  | 46.7 | 46.7 |  | 44.8 | 48.4 |  | 44.8 | | 48.3 |  | 31 | | 62.1 |  | 51.7 | | 41.4 |  |
|  |  |  |  |  |  |  |  |  |  |  |  |  |  |  |  |  | |  |  |  | |  |  |  | |  |  |
| Alopecia (-) | 80 | 75.9 | 0.70 | 79.3 | 75.9 | 0.75 | 76.7 | 78.6 | 0.86 | 70 | 86.7 | 0.12 | 75.9 | 80.6 | 0.65 | 72.4 | | 82.8 | 0.35 | 82.8 | | 72.4 | 0.35 | 86.2 | | 69 | 0.12 |
| Alopecia (+) | 20 | 24.1 |  | 20.7 | 24.1 |  | 23.3 | 21.4 |  | 30 | 13.3 |  | 24.1 | 19.4 |  | 27.6 | | 17.2 |  | 17.2 | | 27.6 |  | 13.8 | | 31 |  |
|  |  |  |  |  |  |  |  |  |  |  |  |  |  |  |  |  | |  |  |  | |  |  |  | |  |  |
| Arthritis (-) | 96.7 | 62.1 | 0.001* | 75.9 | 82.8 | 0.52 | 86.7 | 71.4 | 0.15 | 86.7 | 73.3 | 0.2 | 79.3 | 80.6 | 0.9 | 79.3 | | 79.3 | 1.0 | 79.3 | | 79.3 | 1.0 | 82.8 | | 75.9 | 0.52 |
| Arthritis (+) | 3.3 | 37.9 |  | 24.1 | 17.2 |  | 13.3 | 28.6 |  | 13.3 | 26.7 |  | 20.7 | 19.4 |  | 20.7 | | 20.7 |  | 20.7 | | 20.7 |  | 17.2 | | 24.1 |  |
|  |  |  |  |  |  |  |  |  |  |  |  |  |  |  |  |  | |  |  |  | |  |  |  | |  |  |
| Cardiorespiratory (-) | 80 | 86.2 | 0.73 | 86.2 | 79.3 | 0.49 | 83.3 | 82.1 | 0.91 | 76.7 | 86.7 | 0.32 | 79.3 | 83.9 | 0.65 | 86.2 | | 79.3 | 0.49 | 75.9 | | 89.7 | 0.16 | 79.3 | | 86.2 | 0.49 |
| Cardiorespiratory (+) | 20 | 13.8 |  | 13.8 | 20.7 |  | 16.7 | 17.9 |  | 23.3 | 13.3 |  | 20.7 | 16.1 |  | 13.8 | | 20.7 |  | 24.1 | | 10.3 |  | 20.7 | | 13.8 |  |
|  |  |  |  |  |  |  |  |  |  |  |  |  |  |  |  |  | |  |  |  | |  |  |  | |  |  |
| Neuropsychiatric  (-) | 83.3 | 93.1 | 0.42 | 93.1 | 86.2 | 0.67 | 86.7 | 92.9 | 0.67 | 80 | 96.7 | 0.103 | 89.7 | 87.1 | 1.0 | 89.7 | | 89.7 | 1.0 | 89.7 | | 89.7 | 1.0 | 93.1 | | 86.2 | 0.67 |
| Neuropsychiatric (+) | 16.7 | 6.9 |  | 6.9 | 13.8 |  | 13.3 | 7.1 |  | 20 | 3.3 |  | 10.3 | 12.9 |  | 10.3 | | 10.3 |  | 10.3 | | 10.3 |  | 6.9 | | 13.8 |  |
|  |  |  |  |  |  |  |  |  |  |  |  |  |  |  |  |  | |  |  |  | |  |  |  | |  |  |
| AIHA (+) | 66.7 | 75.9 | 0.44 | 65.5 | 75.9 | 0.39 | 76.7 | 64.3 | 0.3 | 66.7 | 73.3 | 0.57 | 72.4 | 67.7 | 0.69 | 79.3 | | 62.1 | 0.15 | 69 | | 72.4 | 0.77 | 82.8 | | 58.6 | 0.04* |
| AIHA (-) | 33.3 | 24.1 |  | 34.5 | 24.1 |  | 23.3 | 35.7 |  | 33.3 | 36.7 |  | 27.6 | 32.3 |  | 20.7 | | 37.9 |  | 31 | | 27.6 |  | 17.2 | | 41.4 |  |
|  |  |  |  |  |  |  |  |  |  |  |  |  |  |  |  |  | |  |  |  | |  |  |  | |  |  |
| Vasculitis (-) | 83.3 | 82.8 | 1.0 | 69 | 96.6 | 0.01* | 83.3 | 82.1 | 1.0 | 80 | 86.7 | 0.49 | 79.3 | 87.1 | 0.5 | 89.7 | | 75.9 | 16 | 75.9 | | 89.7 | 0.16 | 75.9 | | 89.7 | 0.16 |
| Vasculitis (+) | 16.7 | 17.2 |  | 31 | 3.4 |  | 16.7 | 17.9 |  | 20 | 13.3 |  | 20.7 | 12.9 |  | 10.3 | | 24.1 |  | 24.1 | | 10.3 |  | 24.1 | | 10.3 |  |
|  |  |  |  |  |  |  |  |  |  |  |  |  |  |  |  |  | |  |  |  | |  |  |  | |  |  |
| LN (-) | 70 | 72.4 | 0.84 | 62.1 | 79.3 | 0.15 | 63.3 | 78.6 | 0.2 | 56.7 | 86.7 | 0.02* | 65.5 | 77.4 | 0.31 | 72.4 | | 69 | 0.77 | 72.4 | | 69 | 0.77 | 69 | | 72.4 | 0.77 |
| LN (+) | 30 | 27.6 |  | 37.9 | 20.7 |  | 36.7 | 21.4 |  | 43.3 | 13.3 |  | 34.5 | 22.6 |  | 27.6 | | 31 |  | 27.6 | | 31 |  | 31 | | 27.6 |  |
|  |  |  |  |  |  |  |  |  |  |  |  |  |  |  |  |  | |  |  |  | |  |  |  | |  |  |
| Gastrointestinal (-) | 93.3 | 100 | 0.49 | 100 | 93.1 | 0.49 | 96.7 | 96.7 | 1.0 | 100 | 93.3 | 0.49 | 100 | 93.5 | 0.49 | 100 | | 93.1 | 0.49 | 93.1 | | 100 | P=0.49 | 93.1 | | 100 | 0.49 |
| Gastrointestinal (+) | 6.7 | 0 |  | 0 | 6.9 |  | 3.3 | 3.3 |  | 0 | 6.7 |  | 0 | 6.5 |  | 0 | | 6.9 |  | 6.9 | | 0 |  | 6.9 | | 0 |  |

**S1 Table. Comparison between lymphocyte subsets and clinical manifestations.**

*A value of p<0.05 was considered to indicate statistical significance. Data are presented as percentages of patients. In the column, patients were stratified into 2 groups, high and low, based on the median percentage of each subset, and the presence or absence of each clinical presentation in the row. AIHA, autoimmune hemolytic anemia; LN, lupus nephritis; Tregs, regulatory T cells; NK cells, natural killer cells; NKT cells, natural killer T cells.
