# Supplementary material for: Associations of lymphocyte subpopulations with clinical phenotypes and long-term outcomes in juvenile-onset systemic lupus erythematosus
Source: PLoS One. 2022 Feb 7;17(2):e0263536. doi: 10.1371/journal.pone.0263536 (PMC8820627; doi:10.1371/journal.pone.0263536)
Supplement: S2 Table — *A value of p<0.05 was considered to indicate statistical significance. Data are presented as median (25th–75th percentile). Percentages are given with reference to the total number of lymphocytes, except for the percentage of Tregs within CD4+ T cells given with reference to the total number of CD4+ cells. JSLE, juvenile-onset systemic lupus erythematosus; Tregs, regulatory T cells; NK cells, natural killer cells; NKT cells, natural killer T cells. (DOCX) [file pone.0263536.s004.docx]

**S2 Table. Percentages of lymphocyte subsets in the JSLE patients between treatment-naïve and treated patients during longitudinal follow-up.**

| **Lymphocyte subset** | **Group** | **Longitudinal follow-up JSLE (n=34)** | | **p-value** |
| --- | --- | --- | --- | --- |
|  |  | **Active group** | **Inactive group** |  |
| % Tregs | Treatment-naïve | 1.88 (0.96-2.47) | 1.66 (1.09-2.70) | 0.8904 |
|  | Treated | 0.96 (0.38-1.98) | 1.49 (0.86-2.70) | 0.0663 |
| % Tregs with CD4+ cells | Treatment-naïve | 6.21 (4.03-8.15) | 7.81 (4.98-9.59) | 0.2524 |
|  | Treated | 3.85 (3.17-8.96) | 9.19 (5.71-13.01) | 0.0258* |
| % CD4^+^ T cells | Treatment-naïve | 31.23 (26.90-35.45) | 29.91 (20.58-38.26) | 0.4212 |
|  | Treated | 19.92 (16.83-33.06) | 24.81 (22.42-28.85) | 0.4900 |
| % CD8^+^ T cells | Treatment-naïve | 28.23 (21.38-33.14) | 32.55 (31.45-41.98) | 0.0181* |
|  | Treated | 28.43 (22.15-36.42) | 34.21 (30.32-41.67) | 0.0799 |
| % γδ T cells | Treatment-naïve | 3.73 (3.25-6.00) | 3.50 (1.55-5.77) | 0.3303 |
|  | Treated | 3.90 (2.85-6.39) | 4.15 (2.81-6.88) | 0.7099 |
| % NK cells | Treatment-naïve | 5.68 (5.08-7.69) | 4.25 (2.82-8.96) | 0.9316 |
|  | Treated | 6.15 (4.82-11.87) | 10.23 (4.79-17.43) | 0.1956 |
| % NKT cells | Treatment-naïve | 1.54 (0.55-1.83) | 0.77 (0.45-1.54) | 0.8040 |
|  | Treated | 1.29 (0.81-2.62) | 1.99 (1.09-3.31) | 0.5949 |
| % CD19^+^ B cells | Treatment-naïve | 20.03 (12.62-24.62) | 11.24 (6.53-22.11) | 0.0181* |
|  | Treated | 22.13 (13.79-30.82) | 10.85 (7.15-19.58) | 0.0033* |

*A value of p<0.05 was considered to indicate statistical significance. Data are presented as median (25th–75th percentile). Percentages are given with reference to the total number of lymphocytes, except for the percentage of Tregs within CD4^+^ T cells given with reference to the total number of CD4^+^ cells. JSLE, juvenile-onset systemic lupus erythematosus; Tregs, regulatory T cells; NK cells, natural killer cells; NKT cells, natural killer T cells.
