# Supplementary material for: Associations of lymphocyte subpopulations with clinical phenotypes and long-term outcomes in juvenile-onset systemic lupus erythematosus
Source: PLoS One. 2022 Feb 7;17(2):e0263536. doi: 10.1371/journal.pone.0263536 (PMC8820627; doi:10.1371/journal.pone.0263536)
Supplement: S3 Table — Data are presented as median (25th–75th percentile). Tregs percentages are given with reference to the total number of lymphocytes. *A value of p<0.05 was considered to indicate statistical significance, compared between three points, ap<0.05 compared with 3 months, bp<0.05 compared with baseline. JSLE, juvenile-onset systemic lupus erythematosus; LN, lupus nephritis. (DOCX) [file pone.0263536.s005.docx]

**S3 Table. Percentage of regulatory T cells (Tregs) in JSLE patients between treatment-naïve and treated patients during 6-months follow-up.**

| **Tregs** | **Group** | **Number of patients** | **Baseline** | **3 months** | **6 months** | **p-value** |
| --- | --- | --- | --- | --- | --- | --- |
| All JSLE (n=49) | Treatment-naïve | 18 | 1.49 (0.51-2.41) | 0.86 (0.43-1.58) | 2.36 (1.04-3.01) | 0.1280 |
|  | Treated | 31 | 0.71 (0.28-1.53) | 0.54 (0.26-0.92) | 1.01 (0.54-1.75)^a^ | 0.0230* |
| Non-LN (n=32) | Treatment-naïve | 13 | 2.06 (1.24-2.60) | 0.98 (0.40-1.25) | 2.63 (0.67-3.18) | 0.1988 |
|  | Treated | 19 | 0.71 (0.28-1.66) | 0.58 (0.23-1.08) | 1.33 (0.60-2.55)^a^ | 0.0183* |
| LN (n=17) | Treatment-naïve | 5 | 0.24 (0.09-0.61) | 0.74 (0.50-3.11) | 1.32 (1.24-3.38)^b^ | 0.0239* |
|  | Treated | 12 | 0.71 (0.27-1.36) | 0.54 (0.28-0.79) | 0.69 (0.18-1.00) | 0.5580 |

Data are presented as median (25th–75th percentile). Tregs percentages are given with reference to the total number of lymphocytes. *A value of p<0.05 was considered to indicate statistical significance, compared between three points, ^a^p<0.05 compared with 3 months, ^b^p<0.05 compared with baseline. JSLE, juvenile-onset systemic lupus erythematosus; LN, lupus nephritis.
