# Supplementary material for: Associations of lymphocyte subpopulations with clinical phenotypes and long-term outcomes in juvenile-onset systemic lupus erythematosus
Source: PLoS One. 2022 Feb 7;17(2):e0263536. doi: 10.1371/journal.pone.0263536 (PMC8820627; doi:10.1371/journal.pone.0263536)
Supplement: S4 Table — *A value of p<0.05 was considered to indicate statistical significance. Data are presented as median (25th–75th percentile). SLEDAI-2K, systemic lupus erythematosus disease activity index 2000; ESR, erythrocyte sedimentation rate; WBC, white blood cells; ALC, absolute lymphocyte count; DCT, direct Coombs test; IVIG, intravenous immunoglobulin; AIHA, autoimmune hemolytic anemia; RM, clinical remission on therapy. (DOCX) [file pone.0263536.s006.docx]

**S4 Table. Comparison characteristics of juvenile-onset systemic lupus erythematosus patients between achievement and non-achievement of clinical remission on therapy.**

| **Characteristics** | **RM**  **(n=42)** | **Non-RM**  **(n=18)** | **P-value** |
| --- | --- | --- | --- |
|  |  |  |  |
| Age, years | 12.06 (9.45-14.53) | 12.23 (10.56-14.02) | 0.8766 |
| Female sex, n (%) | 35 (83.33) | 18(100) | 0.0911 |
| SLEDAI-2K | 12 (6-17.25) | 14 (8.75-22.25) | 0.2724 |
| C_3_, g/L | 0.62 (0.40-0.95) | 0.58 (0.25-0.71) | 0.1027 |
| C_4_, g/L | 0.10 (0.05-0.18) | 0.06 (0.04-0.10) | 0.1019 |
| ESR, mm/h | 61 (41.25-81.75) | 68.50 (31.00-85.50) | 0.9535 |
| Anti-dsDNA positivity, n (%) | 30 (71.43) | 14 (77.78) | 0.7549 |
| WBC (×10^9^/L) | 5.61 (4.03-10.08) | 4.72 (3.45-7.28) | 0.2880 |
| ALC (×10^9^/L) | 1.53 (0.98-2.02) | 1.12 (0.58-1.81) | 0.0370* |
| Hematocrit (%) | 31.90 (27-35.78) | 36.10 (27.40-38.50) | 0.5080 |
| Platelets (×10^9^/L) | 217.50 (145.00-319.50) | 178.00(116.00-311.00) | 0.5566 |
| DCT positivity, n (%) | 20 (47.62) | 4 (22.22) | 0.0876 |
| Clinical manifestations, n (%) |  |  |  |
| Fever | 14 (33.33) | 5 (27.78) | 0.7683 |
| Mucosal ulcer | 18 (42.86) | 10 (55.56) | 0.4087 |
| Skin involvement | 24 (57.14) | 13 (72.22) | 0.3866 |
| Alopecia | 8 (19.05) | 5 (27.78) | 0.5035 |
| Arthritis | 9 (21.43) | 3 (16.67) | >0.9999 |
| Serositis | 6 (14.29) | 5 (27.78) | 0.2790 |
| Neuropsychiatric lupus | 5 (11.90) | 2 (11.11) | >0.9999 |
| Lupus nephritis | 7 (16.67) | 10 (76.92) | 0.0001* |
| Vasculitis | 7 (16.67) | 3 (16.67) | >0.9999 |
| AIHA | 15 (35.71) | 3 (16.67) | 0.2196 |

*A value of p<0.05 was considered to indicate statistical significance. Data are presented as median (25th–75th percentile). SLEDAI-2K, systemic lupus erythematosus disease activity index 2000; ESR, erythrocyte sedimentation rate; WBC, white blood cells; ALC, absolute lymphocyte count; DCT, direct Coombs test; IVIG, intravenous immunoglobulin; AIHA, autoimmune hemolytic anemia; RM, clinical remission on therapy.
